# A dialect geography in Yogyakarta-Surakarta isolect in Wedi District: An examination of permutation and phonological dialectometry as an endeavor to preserve Javanese language in Indonesia

**DOI:** 10.1016/j.heliyon.2021.e07660

**Published:** 2021-07-24

**Authors:** Kundharu Saddhono, Wido Hartanto

**Affiliations:** a Universitas Sebelas Maret, Surakarta, Indonesia; b STKIP Al Hikmah, Surabaya, Indonesia

**Keywords:** Geographic dialect, Isolect, Yogyakarta-Surakarta dialect, Diversity, Border area, Permutation and dialectometry

## Abstract

This study reveals the status of Javanese isolect in Wedi District, Klaten Regency which is influenced by Yogyakarta and Surakarta isolect. This is a quantitative descriptive study. Our data was collected from the residents of Wedi District, Klaten. The data was collected from 15 points of observation areas (villages) based on a dialectometric triangle. It includes the speech of local people. We employed the listening and speaking method. The listening method was equipped with note taking and tapping techniques, while the speaking method was equipped with by probing, face-to-face, and recording techniques. The techniques to analyze our data included reciprocal understanding, dialectometry, permutation and isogloss file. Based on our analysis, we find that the isolect status is based on different vocabularies, speeches, subdialects and dialects. This shows that the language diversity in the typical isolect still exists. These findings also prove that the Yogyakarta and Surakarta dialect holds isolect diversity as a representation and evidence of language diversity.

## 1 Introduction

Javanese language is one among thousands of languages in the world that is spoken by approximately 75.5 million people (Conners and Vander Klok, 2016; Nadar, 2007). With such number of speakers, Javanese language is set on 14th most language spoken in the world. In addition to the number of speakers who are generally located on Java, it is also spoken by Javanese communities in Sumatra, Borneo and other islands in Indonesia (Allen, 2013; Ananta et al., 2016; Aryanti, 2015; Ningsih and Harahap, 2019). Furthermore, there are also several communities of Javanese speakers abroad, such as in Suriname, New Caledonia, and Javanese villages in Malaysia (Kaur, 2018; Laurence, 2000; Ramele and Yamazaki, 2013; Sekimoto, 1988; Villerius, 2017; Wariyati, 2018).

A study on language in Wedi District, Klaten Regency is conducted as an endeavor to preserve the culture, to develop and enrich the language treasury in the area. Within the linguistics field in particular dialectology, the Javanese language in Klaten presents as a field of linguistic study with its diverse problems (Herawati, 2017; Heriyanto, 2020; Lestari, 2012). Those diversities include a unique speech of Javanese isolect in the western region (with influence from variations of Jogjakarta dialect) and in the eastern region (with the influence of variations of Solo dialect). This study took place in Wedi District, Klaten Regency. Wedi District shares its borders on all four sides: on the west with Jogonalan District, on the east with Bayat District, on the south with Gantiwarno District, and on the north with Central Klaten District.

**Figure 1:**
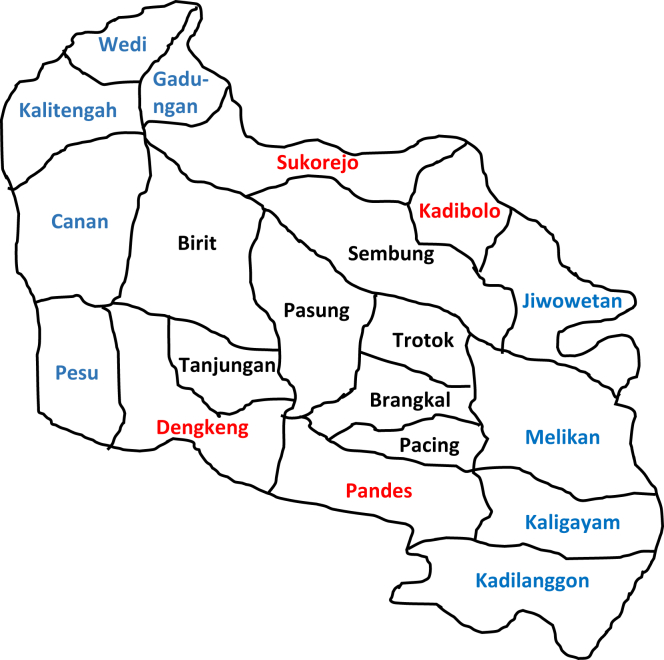
Map of Wedi District, Klaten Regency, Central Java. (Source: original documentation courtesy of the author).

Based on the data taken from Klaten Office (2019), Wedi District covers a total area of 24.38 km^2^ and population density of 1,864/km^2^. From administrative point of view, Wedi district is divided into 19 villages wherein the majority of the local's main livelihood is farming as the area covers agricultural potentials. In addition to the local nature that supports the regional economy, tourism is one of their biggest sources of income. This sector is supported by the local natural such as the stretch of southern coast in Klaten Regency, the natural sceneries and other beautiful natural attractions. Like most residents in Klaten, the residents in Wedi District also speak Javanese. However, this is not to say that the speech conversed between one villager to another within the District is exactly the same. As such, the difference will be examined using dialectology. Previous dialectology studies have been conducted using various data sources (see Figure 1).

The study collected the data from seven Research Areas (RAs) in the eastern district of Kebumen. It shows a problem in speech level and linguistic variations. From the point of dialectological view, it describes the isogloss line and the isogloss documentation. In addition, there is no clear description on the status of isolect on the differences of language, dialect, subdialect, or whether it has difference speeches or no different speeches at all. Ngumarno wrote his thesis titled ‘The Javanese Isolect in Ambal District, Kebumen: A dialectological study’ (Ngumarno, 2010). It emphasizes the existence of various Javanese isolect in Ambal. The data covers six research areas. The result shows documentation and depictions of lexical and phonological isogloss.

Based on the above studies that are based on the current linguistic phenomena, the authors were determined to choose Wedi District, Klaten Regency as the subject of the research. The location is also distinguished as the theme as we wrote this thesis. We chose Wedi District for two reasons. First, Wedi and Ambal District have a lot of similarities in their urban planning. Second, there is uniqueness in the language, that is a blended dialect between Yogyakarta and Surakarta dialect. From the point of phonological view, this phenomenon appears unique, and it appears that the geographical condition of isolect in Jogja-Solo dialect region provides certain language variations.

The authors refer to these linguistic phenomena as isolect. The use of this term aims to neutralize a term for a linguistic phenomenon whose status is still unclear (Asnan, 2019; Mahsun, 1995; Nurmisliah et al., 2018; Rozelin and Azlan, 2019). Based on our first observation, all villages in Wedi district influenced by the Jogja dialects are located in the east and south of the region. Villagers with Solo dialect mostly found in the west and north region.

The dialect used in all villages influenced by Jogja dialect tends to use [I] as in [gərIh] while Solo dialect tends use [ε] as in [gərεh]. Such linguistic phenomenon is used as a reference that YS dialect (Yogyakarta-Surakarta) still carries the difference. Such linguistic phenomena within linguistic study are not yet classified that categorize these phenomena as a language, dialect, sub dialect or merely a speech difference (Conners and Vander Klok, 2016; Saddhono, 2018a, Saddhono, 2018b; Vander Klok, 2015; Wijana, 2005). Based on the above notion, the authors refer this linguistic phenomenon as isolect. The use of this term aims to neutralize a linguistic phenomenon whose status is still unclear. We find that Javanese isolect in Wedi (IJW) is very interesting because, not only that from the linguistic point of view it sounds unique, but also the area of this isolect is located within YS dialect which is actually still has the difference between Jogja dialect and Solo dialect. Based on its geographical location, it is very possible that we will find this unique isolect. Hence, we hope this study will enrich our language diversity. This study will focus on the phonological level of YS isolect in Wedi district, Klaten Regency. In conclusion, this research aims to acknowledge the dialect status in Wedi District using dialectometry numeration.

According to Lauder, dialectology is a subfield in language science that systematically deals with various studies relating to the distribution of dialects or variations in language by taking into account geographic, political, economic, and social cultural factors (Lauder, 2006). Dialectology is also often referred to as a geographical linguistics, geolinguistics, or area linguistics (Anderwald and Szmrecsanyi, 2009). The objective of this study is to use a dialectological approach, namely to identify the status of Javanese isolect under the phonological level in Wedi District, Klaten regency.

## 2 Research method

This is a quantitative study because we used the data and applies computational analyses within dialectology and permutation (Wieling and Nerbonne, 2015). This study was presented using a descriptive technique. The use of this technique allowed the authors to present the facts of language concerning Javanese language isolect in Wedi District. Based on the linguistic facts, we collected the data for further selection and then mapped it. The technique was employed to provide explanation, interpretation, analysis and data processing.

This study is based the dialectology approach (Bailey et al., 1997; Thomas, 1987). Data for the study was collected from the residents in Wedi District, Klaten regency. It includes the residents' normal speech. Based on 19 villages in Wedi District, 15 villages were designated as the observation areas based on a dialectometric triangle pattern that interconnected RA (research area). Those villages include Canan Village (DP 1), Kalitengan Village (DP 2), Gadungan Village (DP 3), Pandes Village (DP 4), Dengkeng Village (DP 5), Pesu Village (DP 6), Birit Village (DP 7), Pasung Village (DP 8), Trotok Village (DP 9), Sukorejo Village (DP 10), Kadibolo Village (DP 11), Sembung Village (DP 12), Melikan Village (DP 13), Jiwowetan Village (DP 14) and Kadilanggon Village (DP 15). We compared all RAs for their respective isolect status based on the percentages of dialectometry and permutation calculation. Equally important was the informants who were determined based on the age criteria i.e. at least 40 years old, never traveled abroad, and still had a complete sense of speech. In addition, Nothofer list of questions has helped the authors to collect the linguistic data that were truly reasonable during the time of speaking. There were 838 glosses and we used a few sentences as the instruments to collect the data needed (Adelaar, 2017; Nothofer, 2007).

The data of Javanese isolect in Wedi District were collected by utilizing the listening and speaking methods (Mahsun, 1995; Thomas, 1988). Both methods have their own techniques. The speaking method allowed the authors to conduct face to face meeting and to hold a conversation with the informants. Consequently, contacts/meetings took place between the authors and the informants in every predetermined RA. The listening method allowed the authors to listen to the use of informant's language, especially the oral communication. This method was conducted by recording the conversation, i.e. recording the language used by the informants in collecting the data.

Technically, the speaking methods were taken using a probing technique. The authors set an inducement to the informant so the informants might produce the expected targeted language. The trick was in the form of gloss prepared and arranged earlier in a list of questions (Filppula et al., 2005). Probing techniques serves as a basic technique done in an advanced technique in the form of advanced conversational techniques. This technique requires the authors to visit every RA and to converse with the informants. In addition to face-to-face technique, the authors also employed an advanced technique e.g. recording. The recording technique was used to complete the notes on the contents of each gloss. One of the problems we had was time limitation. This was because most of our informants worked as farmer. Consequently, the authors had to visit every RA several times. The data collection phase was completed when the data needed was available with clarification (both lexical and phonological). The next phase was data analysis. In practice, there were several methods that might be used in dialectological study i.e. the method of mutual understanding, lexicostatistic, diometrics, Homals and isogloss documentation (Mahsun, 1995; Chambers and Trudgill, 1998).

In this study, the authors analyzed the data using a method of mutual understanding, a dialectometry method to determine the vocabulary distance between one AR and another, and to find the differences or similarities on their isolect status between ARs compared. The method's limitation will be then complemented with permutation method, and isogloss documentation. The three methods were basically related to one another in data analysis. Eventually we found out the status of Javanese isolects in Wedi district. We used the criteria for determining isolect to be defined as language, dialect, sub dialect, or no difference based on Seguy formula (Lauder, 1993; Trudgill, 1986).

$$\frac{\left(s\times 100\right)}{n}=d\%$$

Note:

S: The number of differences compared to other observation areas

n: The number of maps compared

d: The distance of vocabulary in percentage

The findings are presented in percentage of vocabulary from the RAs which determine the status of the isolect with the following criteria:

Phonological differences

17 % and above: considered as language difference

12–16 %: considered as dialect difference

4–11 %: considered as sub-dialectal difference

4–7 %: considered as speech difference

0–3 % and below: considered as no difference

## 3 Results and discussion

### 3.1 The result from dialectometry isolects calculation in Wedi District based on phonological analysis

The phonological dialectometric results were done based on the data which were collected at Wedi District. The data were gathered from the questions in the form of gloss from Nothofer. The data showed that there were phonological differences which were calculated using Seguy Formula. Based on phonological calculation of dialectometry in Wedi District, the results were shown at Table 1 as follow.

**Table 1:** Integrating the results of phonological calculations.

DP No.	%	Isolect Status	DP No.	%	Isolect Status
1–2	6 %	BW	7–10	5.5 %	BW
1–3	7 %	BW	8–9	6 %	BW
2–3	9 %	BS	8–10	7.5 %	BW
2–7	11 %	BS	8–11	7 %	BW
3–4	11.5 %	BS	9–15	5.5 %	BW
3–5	9 %	BS	10–11	8 %	BS
3–7	7.5 %	BW	10–12	8 %	BS
4–5	6 %	BW	11–9	8 %	BS
5–6	7 %	BW	11–12	8.5 %	BS
5–7	14.5 %	BD	12–9	6 %	BW
5–8	8.5 %	BS	13–9	6.5 %	BW
6–8	2.5 %	BK	13–12	16 %	BD
6–9	2 %	BK	13–14	7.5 %	BW
6–15	3 %	BK	14–9	5.5 %	BW
7–8	6 %	BW	14–15	5 %	BW

**Note:**

BW (Difference in Speech).

BS (Difference in Sub dialect).

BD (Difference in Dialect).

As shown in Table 1, if there are different isolation statuses in one column, the highest status will be used by the authors. Based on the result of the phonological calculation in Table 1, a map of polygon phonology can be created. The map is presented to illustrate a visualization to understand the relationship status among- RAs isolect which is symbolized by different thicknesses of lines and color. The thinnest black line represents the differences in vocabulary, the thinner red lines represents the differences in speech, the thicker yellow line represents subdialect differences, and the thick blue line represents the dialect differences.

**Figure 2:**
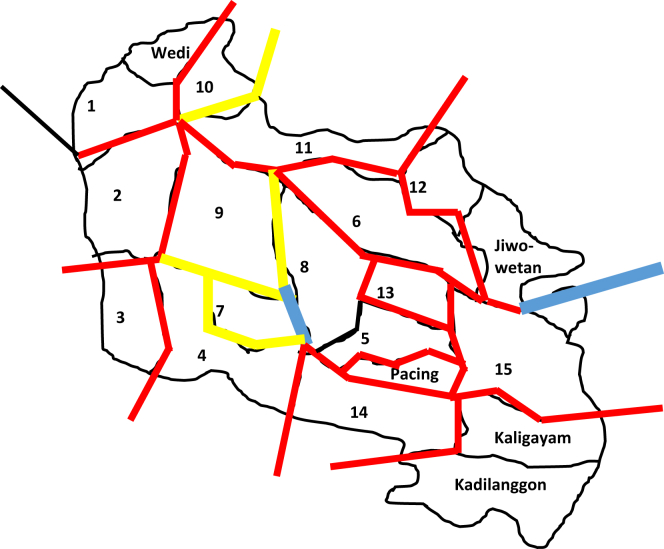
Map of Phonological Dialectometry. (Source: original documentation courtesy of the author). Description: : Difference in Vocabulary : Difference in Speech : Difference in Subdialect : Difference in Dialect.

The Figure 2 shows that there are two dialect border lines found in RA 5–7, and 12–13. This means that there are two RAs whose isolects have the status of dialect, namely RA 5–7, and 12–13 based on the dialectometric patterns. The rest of RAs have different vocabulary statuses, different speeches and different subdialects. RA 5–7 is geographically separated by a large river and a small bridge, therefore interaction among those residents was hampered. RA 12–13 is separated by a small hill which geographically makes the residents in the second RA cannot directly interact with one another.

Based on the mapping of dialectometry in phonology, we can state the term Dengkeng, Birit, Kadipolo and Jiwowe dialect. Dengkeng dialect is a term for the isolect found in RA 5 in relation to RA 7 isolect. The relationship between the two isolects is that they are of different dialect, hence RA 7 is set as a distinctive dialect, in this case, we call it Birit dialect. RA 12 and 13 are called Kadibolo and Jiwowetan dialect status respectively.

### 3.2 The result of phonological permutation calculation

Based on the calculation of phonological permutation of Wedi District, if we find two statuses, we will apply the highest status. The following table calculates the isolect phonological permutation in Wedi District.

Based on the calculation, we found that there are six different vocabulary words, 44 different speech lines, 51 different sub-dialects and four different dialects. The calculation also explains other RA status that was not in the lexical and phonological dialectometry. Table 2 also shows that the status of speech difference and subdialect difference is more dominant compared to other statuses.

**Table 2:** Phonological permutations.

DP No.	%	Status	DP No.	%	Status	DP No.	%	Status
1–2	6	BW	3–12	8	BS	7–9	7.5	BW
1–3	7	BW	3–13	7	BW	7–10	5.5	BW
1–4	11.5	BS	3–14	6.5	BW	7–11	5.5	BW
1–5	12	BD	3–15	6.5	BW	7–12	8.5	BS
1–6	9.5	BS	4–5	6	BW	7–13	8.5	BS
1–7	9.5	BS	4–6	8	BS	7–14	4	BW
1–8	7.5	BW	4–7	15.5	BD	7–15	9	BS
1–9	9.5	BS	4–8	11	BS	8–9	6	BW
1–10	8	BS	4–9	7	BW	8–10	7.5	BW
1–11	10.5	BS	4–10	7.5	BW	8–11	7	BW
1–12	8	BS	4–11	6	BW	8–12	6.5	BW
1–13	9	BS	4–12	6	BW	8–13	6	BW
1–14	10.5	BS	4–13	8	BS	8–14	6.5	BW
1–15	9.5	BS	4–14	10	BS	8–15	6.5	BW
2–3	9	BS	4–15	9	BS	9–10	9	BS
2–4	9	BS	5–6	7	BW	9–11	8	BS
2–5	10.5	BS	5–7	14.5	BD	9–12	6	BW
2–6	4	BW	5–8	8.5	BS	9–13	6.5	BW
2–7	11	BS	5–9	8.5	BS	9–14	7	BW
2–8	2.5	BK	5–10	7.5	BW	9–15	5	BW
2–9	2	BK	5–11	10	BS	10–11	8	BS
2–10	4.5	BW	5–12	10	BS	10–12	8	BS
2–11	5	BW	5–13	4.5	BW	10–13	7	BW
2–12	3	BK	5–14	9	BS	10–14	8	BS
2–13	10	BS	5–15	10.5	BS	10–15	6	BW
2–14	6.5	BW	6–7	5	BW	11–12	8.5	BS
2–15	6	BW	6–8	2.5	BK	11–13	10	BS
3–4	11.5	BS	6–9	2	BK	11–14	4	BW
3–5	9	BS	6–10	4	BW	11–15	10.5	BS
3–6	8.5	BS	6–11	4.5	BW	12–13	16	BD
3–7	7.5	BW	6–12	10.5	BS	12–14	10	BS
3–8	10	BS	6–13	6.5	BW	12–15	5.5	BW
3–9	7.5	BW	6–14	5.5	BW	13–14	7.5	BW
3–10	8.5	BS	6–15	3	BK	13–15	6.5	BW
3–11	10	BS	7–8	6	BW	14–15	5	BW

**Note:**

BK (Different Vocabulary).

BW (Different Speech).

BS (Different Sub dialect).

BD (Different Dialect).

### 3.3 The description of phonological differences

The followings are presentation of phonological differences found in each RA. There are differences in two phonemes and also differences in three phonemes. However, all of them are dominated by differences in two phonemes that appear in every RA. In general, 104 phonological differences were found in every RA. These differences include differences in the vocal and consonants. Phonological differences have the same position as lexical differences in determining the status of isolect. Phonological and lexical differences produce the percentage which, in the later stage, determines the status of isolect in each RA.

#### 3.3.1 Correspondence of sound -a- ~ -ə-

Table 3 presents the correspondence analysis results of sound -a- ~ -ə-

**Table 3:** Correspondence of sound -a- ~ -ə-.

No.	Gloss (Number)	Affix I Using -a-	Affix II Using -ə-
1.	*Dahi* [forehead] (12)	*Palarapan* *Paraupan*	*pəlarapan* *pəraupan*
2.	*Jari tengah* [middle finger] (28)	*panuŋgUl*	*pənuŋgUl*
3.	*Pelupuk mata* [eyelid] (55)	*talapU?an*	*təlapU?an*
4.	*Telapak kaki* [food sole] (70)	*dalama?an* *tapa?an*	*dəlama?an* *təpa?an*
5.	*Anak yang tertua* [oldest/eldest child] (110)	*pambarəp*	*pəmbarəp*
6.	*Istri adik laki-laki ayah* [father's younger sister in law] (116)	*Paripεn*	*pəripεn*
7.	*Istri adik laki-laki ibu* [mother's younger sister in law](117)	*Paripεn*	*pəripεn*
8.	*Istri kakak laki-laki ayah* [father's older sister in law] (118)	*Paripεn*	*pəripεn*
9.	*Istri kakak laki-laki ibu* [mother's older sister in law] (119)	*Paripεn*	*pəripεn*
10.	*Istri dari saudara* [brother's wife] (120)	*Paripεn*	*pəripεn*
11.	*Saudara dari istri* [wife's brother/sister] (137)	*Paripεn*	*pəripεn*
12.	*Saudara dari suami* [husband's brother/sister] (138)	*Paripεn*	*pəripεn*
13.	*Suami adik perempuan ayah* [father's younger sister's husband] (139)	*Paripεn*	*pəripεn*
14.	*Suami adik perempuan ibu* [mother's younger sister's husband] (140)	*Paripεn*	*pəripεn*
15.	*Suami dari saudara* [sister's husband] (141)	*Paripεn*	*pəripεn*
16.	*Suami dari istri saudara istri* [wife's sister's husband] (143)	*Paripεn*	*pəripεn*
17.	*Suami/istri saudara suami* [husband's brother/sister's spouse] (144)	*Paripεn*	*pəripεn*
18.	*Suami kakak perempuan ayah* [father's older sister's husband] (146)	*Paripεn*	*pəripεn*
19.	*Suami kakak perempuan ibu* [mother's older sister's husband] (147)	*Paripεn*	*pəripεn*
20.	*Kandang merpati* [Dove House] (192)	*Pagupכn*	*pəgupכn*
21.	*Langit-langit* [Ceiling] (195)	*Palapכn*	*pəlapכn*
22.	Ruang depan yang terbuka [*Open Front Room*] (204)	*Pariŋgitan*	*pəriŋgitan*
23.	*Setandan pisang* [A Bunch of Banana] (350)	*satundUn gəDaŋ*	*sətundUn gəDaŋ*
24.	*Sisir pisang* [A Comb of Banana ] (351)	*Saliraŋ*	*səliraŋ*
25.	*Jalan sempit* [Small Path] (460)	*Paluran*	*pəluran*
26.	*Jurang* [Ravine] (461)	*Jarambaŋan*	*jərambaŋan*
27.	*Utara* [North] (502)	*Alכr*	*əlכr*
28.	*Membawa dengan ketiak* [Bringing something using armpit] (557)	*ŋampIt*	*ŋəmpIt*
29.	*Membawa di pinggang* [bringing something around waist] (564)	*ňaŋklε(?)*	*ňəŋklε(?)*
30.	*Memburu hewan malam* [hunting animals at night] (570)	*Ramaŋan*	*rəmaŋan*
31.	*Sebentar* [ waiting for a while] (489)	*saDəlכ?*	*səDəlכ?*
32.	*Mencium benda* [smelling something] (583)	*ambu*	*ŋəmbu*
33.	*Menganyam* [weaving] (589)	*aňam*	*ŋaňəm*
34.	*Menyuruh* [ordering] (620)	*akכn*	*ŋəkכn*
35.	*Buta* [blind] (743)	*pica?*	*picə?*
36.	*Sejengkal* [a span of hand] (765)	*Sakilan*	*səkilan*

#### 3.3.2 Correspondence of sound -ε- ~ -i-

Table 4 shows the correspondence analysis results of sound -ε- ~ -i-

**Table 4:** Correspondence of sound -ε- ~ -i-.

No.	Gloss (Number)	Affix I Using -ε-	Affix II Using -i-
1.	*Darah* [blood] (13)	*gətεh*	*gətih*
2.	*Sebentar* [waiting for a while] (489)	*Delεt*	*Delit*
3.	*Tebing* [cliff] (497)	*Pεrεŋan*	*Piriŋan*
4.	*Banyak* [a lot] (653)	*Akεh* *wakεh* *wuakεh*	*Akih* *wakih* *wuakih*
5.	*Pendek* [short] (708)	*cənDε?*	*cənDi?*
		*c(u)ənDε?*	*c(u)ənDi?*
		*pənDε?*	*pənDi?*
		*p(u)ənDε?*	*p(u)ənDi?*
6.	*Rendah* [low] (714)	*cənDε?* *c(u)ənDε?* *ənDε?* *(u)ənDε?*	*cənDi?* *c(u)ənDi?* *ənDi?* *(u)ənDi?*
7.	*Ringan* [light] (715)	*Εntεŋ* *(u)εntεŋ*	*Εntiŋ* *(u)εntiŋ*
8.	*Takut* [afraid] (723)	*Jirεh*	*Jirih*

#### 3.3.3 Correspondence of sound -i- ~ -I-

Table 5 presents the results of correspondence analysis of sound -i- ~ -I-

**Table 5:** Correspondence of sound -i- ~ -I-.

No.	Gloss (Number)	Affix I Using -i-	Affix II Using -I-
1.	*Bersih* [clean] (660)	*resi?* *r(u)esi?*	*resI?* *r(u)esI?*
2.	*Jernih* [clear] (683)	*bəniŋ* *b(u)əniŋ*	*bənIŋ* *b(u)ənIŋ*
3.	*Kecil* [small] (685)	*cili?* *c(u)ili?*	*cilI?* *c(u)ilI?*
4.	*Kering* [dry] (688)	*Gariŋ* *Gariŋ*	*garIŋ* *garIŋ*
5.	*Kikir* [stingy] (689)	*uTil*	*uTIl*
6.	*Malu* [shy] (698)	*Isin* *i(u)sin*	*isIn* *i(u)sIn*
7.	*Tajam* [sharp] (722)	*Lancip* *l(u)ancip* *lincip* *l(u)incip*	*lancIp* *l(u)ancIp* *lincIp* *l(u)incIp*
8.	*Tipis* [thin] (732)	*Tipis* *t(u)ipis*	*tipIs* *t(u)ipIs*

#### 3.3.4 Correspondence of sound -a- ~ -ε-

Table 6 shows the correspondence analysis results of sound -a- ~ -ε-

**Table 6:** Correspondence of sound -a- ~ -ε-.

No.	Gloss (Number)	Affix I Using -a-	Affix II Using -ε-
1.	*Di atas* [on/above] (437)	*naŋ (n)duwUr*	*nεŋ (n)duwUr*
2.	*Di bawah* [under/beneath] (438)	*naŋ ŋisכr*	*nεŋ ŋisכr*
3.	*Di sana* [there] (440)	*naŋ kכnכ*	*nεŋ kכnכ*
4.	*Di sini* [here] (441)	*naŋ kene*	*nεŋ kene*
5.	*Tertawa* [laughing] (641)	*ŋaka?*	*ŋεkε?*
6.	*Pendek* [short] (702)	*cənDa?*	*cənDε?*

#### 3.3.5 Correspondence of sound -a- ~ -i-

Table 7 shows the correspondence analysis results of sound -a- ~ -i-

**Table 7:** Correspondence of sound -a- ~ -i-.

No.	Gloss (Number)	Affix I Using -a-	Affix II Using -i-
1.	*Cabai merah* [red chili] (307)	*lכmbכ? Abaŋ*	*lכmbכ? Abiŋ*
2.	*Dekat* [near] (671)	*cəDa?*	*cəDi?*
3.	*Merah* [red] (702)	*Abaŋ*	*Abiŋ*
4.	*Pendek* [short] (708)	*cənDa?*	*cənDi?*
5.	*Tajam* [sharp] (722)	*lancIp*	*lincIp*
6.	*Terang* [bright] (723)	*Padaŋ*	*Padiŋ*

#### 3.3.6 Correspondence of sound -u- ~ -U-

Table 8 presents the correspondence analysis results of sound -u- ~ -U-

**Table 8:** Correspondence of sound -u- ~ -U-.

No.	Gloss (Number)	Affix I Using -u-	Affix II Using -U-
1.	*Bulu roma* [skin hairs] (8)	*rambUt alus*	*rambUt alUs*
2.	*Fajar* [dawn] (449)	*esu?*	*esU?*
3.	*Pagi* [morning] (483)	*esu?*	*esU?*
4.	*Pagi sekali* [early morning] (484)	*esu?*	*esU?*
5.	*Halus* [soft] (678)	*Alus*	*alUs*
6.	*Tinggi gunung* [(a mountain) high] (730)	*Duwur*	*duwUr*
7.	*Tinggi orang* [(a man) tall] (731)	*Duwur*	*duwUr*

#### 3.3.7 Correspondence of sound -n- ~ -ň-

Table 9 shows the correspondence analysis results of sound -n- ~ -ň-

**Table 9:** Correspondence of sound -n- ~ -ň-.

No.	Gloss (Number)	Affix I Using -n-	Affix II Using -ň-
1.	*Di sana* [there] (440)	*naŋ kכnכ*	*ňaŋ kכnכ*
2.	*Di sini* [here](441)	*naŋ kene*	*ňaŋ kene*
3.	*Di atas* [on/above] (437)	*naŋ nduwUr*	*ňaŋ nduwUr*
4.	*Di bawah* [under/beneath] (438)	*naŋ ŋisכr*	*ňaŋ ŋisכr*
5.	*Menyusul* [catching up] (621)	*nusUl*	*ňusUl*

#### 3.3.8 Correspondence of sound -b- ~ -g-

Table 10 is the correspondence analysis results of sound -b- ~ -g-

**Table 10:** Correspondence of sound -b- ~ -g-.

No.	Gloss (Number)	Affix I Using -b-	Affix II Using -g-
1.	*Geraham* [molars] (14)	*Bam*	*Gam*
2.	*Kemaluan perempuan* [vagina] (441)	*bawU?*	*gawU?*
3.	*Jatuh daun, buah, dan lain-lain* [falling leaf, fruit, and others] (537)	*cəblכ?* *jəblכ?* *ciblכ?* *jiblכ?*	*cəglכ?* *jəglכ?* *ciglכ?* *jiglכ?*

#### 3.3.9 Correspondence of sound -i- ~ -ə-

Table 11 is the correspondence analysis results of sound -i- ~ -ə-

**Table 11:** Correspondence of sound -i- ~ -ə-.

No.	Gloss (Number)	Affix I Using -i-	Affix II Using -ə-
1.	*Benih* [seed] (294)	*winIh*	*wənIh*
2.	*Jatuh daun, buah, dan lain-lain* [falling leaf, fruit, and others] (537)	*Rigכl*	*rəgכl*
3.	*Melihat* [seeing] (547)	*ŋiŋəti*	*ŋəŋəti*

#### 3.3.10 Correspondence of sound -c- ~ -j-

Table 12 is the correspondence analysis results of sound -c- ~ -j-

**Table 12:** Correspondence of sound -c- ~ -j-.

No.	Gloss (Number)	Affix I Using -c-	Affix II Using -j-
1.	*Lutut* [knee] (44)	*cəŋkU?*	*jəŋkU?*
2.	*Jendela* [window] (184)	*cəndelכ*	*jəndelכ*
3.	*Jatuh daun, buah, dan lain-lain* [falling leaf, fruit, and others] (537)	*ciblכ?* *cəblכ?* *ciglכ?* *cəglכ?*	*jiblכ?* *jəblכ?* *jiglכ?* *jəglכ?*

#### 3.3.11 Correspondence of sound -o- ~ -u-

Table 13 is the correspondence analysis results of sound -o- ~ -u.

**Table 13:** Correspondence of sound -o- ~ -u.

No.	Gloss (Number)	Affix I Using -o-	Affix II Using -u-
1.	*Cabai hijau* [green chili] (306)	*lכmbכ? ijo*	*lכmbכ? Iju*
2.	*Hijau* [green] (681)	*Ijo*	*Iju*
3.	*Kutang* [bra] (767)	*Kotaŋ*	*Kutaŋ*

#### 3.3.12 Correspondence of sound -e- ~ -i-

The followings are the correspondence analysis results of sound -e- ~ -i- (see Table 14).

**Table 14:** Correspondence of sound -e- ~ -i-.

No.	Gloss (Number)	Affix I Using -e-	Affix II Using -i-
1.	*Besar* [big] (661)	*gəDe*	*gəDi*
2.	*Lama* [long] (694)	*Suwe*	*Suwi*
3.	*Tajam* [sharp] (722)	*Lancep*	*Lancip*

#### 3.3.13 Correspondence of sound -u- ~ -כ-

Table 15 shows the correspondence analysis results of sound -u- ~ -כ-

**Table 15:** Correspondence of sound -u- ~ -כ-.

No.	Gloss (Number)	Affix I Using -u-	Affix II Using -כ-
1.	*Jauh* [far] (682)	*aDuh* *wuaDuh*	*aDכh* *wuaDכh*
2.	*Lama* [long] (694)	*Dכwu* *Duכwu*	*Dכwכ* *Duכwכ*
3.	*Sakit* [sick] (717)	*lכru*	*Lכrכ*

#### 3.3.14 Correspondence of sound -i- ~ -I- ~ -ε-

Table 16 is the correspondence analysis results of sound -i- ~ -I- ~ -ε-

**Table 16:** Correspondence of sound -i- ~ -I- ~ -ε-.

No.	Gloss (Number)	Affix I Using -i-	Affix II Using -I-	Affix II Using -ε-
1.	*Pendek* [short] (708)	*cənDi?*	*cənDI?*	*cənDε?*
2.	*Lama* [long] (714)	*(c)ənDi?*	*(c)ənDI?*	*(c)ənDε?*
3.	*Takut* [afraid] (723)	*Jirih*	*jirIh*	*Jirεh*

#### 3.3.15 Correspondence of sound -ə- ~ -u-

Table 17 shows the correspondence analysis results of sound -ə- ~ -u-

**Table 17:** Correspondence of sound -ə- ~ -u-.

No.	Gloss (Number)	Affix I Using -ə-	Affix II Using -u-
1.	*Sayap* [wing] (396)	*səwiwi*	*Suwiwi*
2.	Berkembang pohon [flowering tree](516)	*əwכh*	*Uwכh*

#### 3.3.16 Correspondence of sound -i- ~ -u-

Table 18 shows the correspondence analysis results of sound -i- ~ -u-

**Table 18:** Correspondence of sound -i- ~ -u-.

No.	Gloss (Number)	Affix I Using -ə-	Affix II Using -u-
1.	*Sebentar* [waiting for a while] (489)	*dilit*	*Dilut*
2.	*Turun* [getting down] (645)	*midUn*	*mudUn*

#### 3.3.17 Correspondence of sound -ŋ- ~ -m-

Table 19 shows the correspondence analysis results of sound -ŋ- ~ -m-

**Table 19:** Correspondence of sound -ŋ- ~ -m-.

No.	Gloss (Number)	Affix I Using -ŋ-	Affix II Using -m-
1.	*Melihat* [seeing] (547)	*ŋəŋəti*	*nəməti*
2.	*Melirik* [seeing at glance] (548)	*ŋlirI?*	*mlirI?*

#### 3.3.18 Correspondence of sound -d- ~ -j-

Table 20 shows the correspondence analysis results of sound -d- ~ -j-

**Table 20:** Correspondence of sound -d- ~ -j-.

No.	Gloss (Number)	Affix I Using -d-	Affix II Using -j-
1.	*Dada* [chest] (9)	*DכDכ*	*Jכjכ*
2.	*Rusa* [deer] (395)	*Kidaŋ*	*Kijaŋ*

#### 3.3.19 Correspondence of sound -p- ~ -k-

Table 21 shows the correspondence analysis results of sound -p- ~ -k-

**Table 21:** Correspondence of sound -p- ~ -k-.

No.	Gloss (Number)	Affix I Using -p-	Affix II Using -k-
1.	*Asap* [smoke] (419)	*pəlU?*	*kəlU?*
2.	*Kabut* [fog] (462)	*pəDut*	*kəDut*

#### 3.3.20 Correspondence of sound -b- ~ -w-

Table 22 shows the correspondence analysis results of sound -b- ~ -w-

**Table 22:** Correspondence of sound -b- ~ -w-.

No.	Gloss (Number)	Affix I Using -b-	Affix II Using -w-
1.	*Malam* [night] (473)	*bəŋi*	*wəŋi*
2.	*Bergerak* [moving] (511)	*Obah*	*Owah*

#### 3.3.21 Correspondence of sound -a- ~ -כ-

Table 23 shows the correspondence analysis results of sound -a- ~ -כ-

**Table 23:** Correspondence of sound -a- ~ -כ-.

No.	Gloss (Number)	Affix I Using -b-	Affix II Using -w-
1.	*Menggigit manusia* [biting a man] (594)	*bəŋi*	*wəŋi*
2.	*Menggigit serangga* [biting an insect] (595)	*Obah*	*Owah*

#### 3.3.22 Correspondence of sound -p- ~ -m-

Table 24 shows the correspondence analysis results of sound -p- ~ -m-

**Table 24:** Correspondence of sound -p- ~ -m-.

No.	Gloss (Number)	Affix I Using -p-	Affix II Using -m-
1.	*Menjemur* [dried in the sun] (618)	*ŋəpe* *mepe* *pepe* *peme*	*ŋəme* *meme* *meme* *meme*

#### 3.3.23 Correspondence of other sounds

Table 25 shows the correspondence analysis results of other sounds.

**Table 25:** Correspondence of other sounds.

No.	Gloss (Number)	Affix I	Affix II
1.	Pelipis [temple] (54) -l- ~ -r-	*piliŋan*	*Piriŋan*
2.	Bertunangan [Engaging] (150) -n- ~ -t-	*nəŋəri*	*təŋəri*
3.	Tempat tungku [hearth fire] (211) -o- ~ -כ-	*Pogo*	*Pכgכ*
4.	Darat [land] (434) -e- ~ -ε-	*pəDen*	*pəDεn*
5.	Bertanya [questioning] (525) -a- ~ -e-	*takכ(?)*	*tekכ(?)*
6.	Melirik [seeing at glance] (548) -i- ~ -כ-	*mliri?*	*mlכrכ?*
7.	Memberi [giving] (567) -m- ~ -w-	*Mεnεhi*	*Wεnεhi*

Based on the findings on isolects in Wedi District, Klaten Regency by the RAs, it could be concluded that the phonological level that shows the differences in vocabulary are located in 6 RAs. The RAs which show differences in speech are found in 15 RAs. The RAs which show differences in subdialect are located in 15 RAs. The RAs that show the differences in dialect are found in 4 RAs.

Based on the previous analysis, there are layers of society (associative stylistics) that determine the speech level used among Wedi residents. The layers are classified into several levels, namely the grass root layer (i.e. lowest layer), the middle layer and the upper layer (top layer) (Dwiraharjo, 2006; Gumperz, 2009; Rampton, 2010). The grass root layer or the lowest layer consists of people whose speeches sound tough. The middle layer consists of people with finer speech and they usually work as students, teachers or other professions with higher education background. The upper layer consists of people with refined speech and usually include people with high positions, big influence or popular. In reality, although not all generally applied, we endeavor to uncover the extent of social strata that comes from observing all villages studied (Bernstein, 1960; Jansma and Jelsma, 1987).

The result also demonstrates the abundant variations of Javanese language, in particular the speech in Wedi District. Javanese people are diverse both in social, and regional. Findings of this study are also in line with that of Agustina on variations of Javanese language used in the market. During the interaction of buying and selling, Javanese people use a variety of *Ngoko* and *Kromo* styles [46]. Javanese people use both *Ngoko alus* and *naïve Ngoko* styles. On the other hand, Javanese also use *refined Kromo* and *naive Kromo s*tyle. This confirms that Javanese culture has a high degree of variations. The study of Javanese language variations has also been carried out by many authors which demonstrate that Javanese language is rich in meanings and extremely unique in the world (Agustine, 2013; Day, 1978; Mardikantoro and Maretta, 2016; Pujiyanto, 2007).

In general, this study also shows the language diversity within the framework of diversity as an asset of Indonesian people. This is in line with Collins on “Language Diversity and Community Agreements: Plurality and Communication” (Collins, 2014). Collins states the plurality of cultures and ethnicities as well as the diversity of Indonesian languages and 706 regional languages must be perceived as the state and national assets that do not impede communication or national unity. The local languages must be respected and developed simultaneously along with the development of national languages (Cohn and Ravindranath, 2014; Goebel, 2008; Lauder, 2006; Musgrave, 2014; Riza, 2008; Steinhauer, 1992). We shall not let the principles and ideas of Indonesia's diversity sink into framework of imaginary language. As such, the local language will in fact enrich Indonesian language which is increasingly recognized by the international communities through the course of Indonesian language for Foreign Speakers (BIPA).

Findings of this study indicate the phenomenon of linguistic diversity within the framework of diversity into its own wealth for the nation (Goebel, 2018; Saddhono, 2018a, Saddhono, 2018b; Schefold, 1998). The language diversity is full of history. If it does not properly to be managed, it can certainly lead to extinction. Conversely, if the language diversity is managed and promoted properly, it certainly prolongs the breath of culture. Linguistic diversity is one of the reliefs of life in the realm of diversity of the nation.

## 4 Conclusion

The data collected from our respondents (838 glosses) was each sorted into phonological and lexical differences. The details include 104 phonological differences, 32 lexical differences and 9 glosses without differences (zero). Based on the combination of dialectometry calculation as in lexical and phonological, it can be seen that the lowest status is the difference in vocabulary, followed by the difference in speech, the difference in sub dialect, and finally the highest status which is the difference in dialect. Findings from combining permutation calculation of lexical and phonological bear the similarities with the calculation results of lexical and phonological dialectometry. It was found that the lowest status is the difference in vocabulary, followed by the difference in speech, the difference in sub dialect and finally the highest status, namely the difference in dialect. In all areas of observation, there are no different status. The diversity of Javanese isolect linguistics in Wedi district is a real example of a support of diversity. Thus, the linguistic diversity within the framework of diversity should receive a special attention and be promoted into something interesting.

## Declarations

### Author contribution statement

Kundharu Saddhono: Conceived and designed the experiments; Analyzed and interpreted the data; Wrote the paper.

Wido Hartanto: Conceived and designed the experiments; Performed the experiments; Contributed reagents, materials, analysis tools or data; Wrote the paper.

### Funding statement

This work was supported by Universitas 10.13039/501100007690Sebelas Maret Surakarta and STKIP Al Hikmah Surabaya.

### Data availability statement

Data included in article/supplementary material/referenced in article.

### Declaration of interests statement

The authors declare no conflict of interest.

### Additional information

No additional information is available for this paper.
